# Small Renal Cell Carcinoma Presenting With Testicular Metastasis: A Rare Case of pT1a Disease With an Aggressive Clinical Course

**DOI:** 10.7759/cureus.77304

**Published:** 2025-01-11

**Authors:** Mayuka Shinohara, Shinro Hata, Haruto Nishida, Hiromitsu Mimata, Toshitaka Shin

**Affiliations:** 1 Department of Urology, Faculty of Medicine, Oita University, Yufu, JPN; 2 Department of Diagnostic Pathology, Faculty of Medicine, Oita University, Yufu, JPN

**Keywords:** batson venous plexus, contralateral, metastasis, small renal cell carcinoma, testicular tumor

## Abstract

Testicular metastasis of a renal cell carcinoma (RCC) is extremely rare, particularly in the initial clinical presentation. Herein, we describe a unique case in which a small renal mass (pT1a RCC) initially manifested as a contralateral testicular metastasis. A 64-year-old man presented with a right intrascrotal mass. Radiology revealed multiple enlarged retroperitoneal lymph nodes and an 18-mm mass in the left kidney. Following right orchidectomy, the tumor was pathologically not of testicular origin but metastasis. Subsequently, the patient underwent robot-assisted left partial nephrectomy (RAPN) and para-aortic lymph node dissection. Histopathological analysis confirmed RCC with testicular metastasis (pT1aN1M1). Despite administration of two lines of systemic therapy, the patient died of metastatic disease 21 months after RAPN. To the best of our knowledge, this is the first documented case of a pT1a RCC presenting initially as a contralateral testicular metastasis. Our findings highlight the importance of considering metastatic RCC in the differential diagnosis of testicular masses.

## Introduction

Renal cell carcinoma (RCC) accounts for approximately 4% of all adult malignancies [1], with 25-30% of patients presenting with metastatic disease at initial diagnosis [2]. Metastatic RCC typically involves the lungs, lymph nodes, bones, liver, and central nervous system. Testicular metastasis of RCC is exceptionally rare, with only sporadic cases reported in the literature [3]. Herein, we report the first documented case of a small renal mass (pT1a RCC) initially manifesting as contralateral testicular metastasis, which represents a unique presentation in the spectrum of metastatic RCC.

## Case presentation

**Figure 1:**
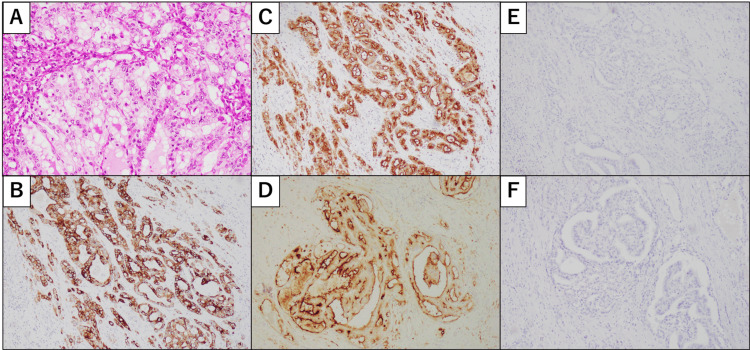
Histopathological findings of testicular metastasis. A: Hematoxylin and eosin (H&E) staining following right orchiectomy shows tumor cells with vesicular round nuclei, pale eosinophilic to clear cytoplasm and a tubular growth pattern with sheet formation (×200). B-F: Immunohistochemistry following right orchidectomy reveals AE1/AE3 (B), epithelial membrane antigen (C), CD10 (D) positivity, renal cell carcinoma (RCC) marker (E), and steroidogenic factor 1 (F) negativity (×100).

A 64-year-old man presented with a right testicular mass. His medical history included diabetes mellitus, hypertension, chronic thyroiditis, and a 45-pack-year smoking history. Serum tumor markers were within normal limits: human chorionic gonadotropin <1.0 IU/L; alpha-fetoprotein, 2.1 μg/L; and lactate dehydrogenase, 156 U/L. The patient underwent right radical high orchidectomy. Immunohistochemical analysis revealed AE1/AE3, epithelial membrane antigen, and CD10 positivity and CK7, CK20, RCC marker, and steroidogenic factor 1 negativity (Figure 1). Because the histopathological findings were inconclusive for a primary testicular neoplasm, the patient was referred to our institution for further evaluation.

**Figure 2:**
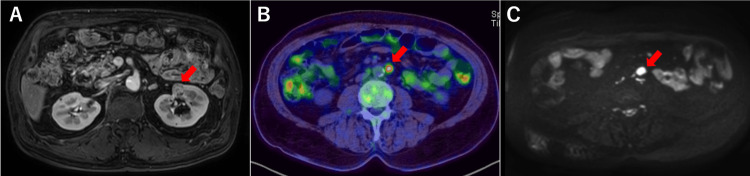
Imaging findings at presentation. A: Computed tomography (CT) shows 24 × 18 mm mass in the upper pole of the left kidney (arrow). B: 18F-fluorodeoxyglucose (FDG)-positron emission tomography/computed tomography reveals multiple enlarged para-aortic lymph nodes with increased FDG uptake (arrow), measuring up to 10 mm in the short axis diameter. C: Diffusion-weighted imaging (DWI) with apparent diffusion coefficient (ADC) mapping showed the restricted diffusion of the corresponding retroperitoneal lymph nodes (arrow).

Computed tomography (CT) revealed multiple retroperitoneal lymph nodes enlarged up to 10 mm in the short-axis diameter and a 24 × 18 mm mass in the upper pole of the left kidney. Magnetic resonance imaging characterized the renal lesion as an 18-mm multilocular cystic mass with contrast enhancement at its superior margin (Figure 2A). Suggestive of malignancy, 18F-fluorodeoxyglucose-positron emission tomography/CT revealed tracer uptake in the retroperitoneal lymph nodes (Figure 2B); there was no significant uptake in the renal mass. Diffusion-weighted imaging (DWI) with apparent diffusion coefficient (ADC) mapping showed the restricted diffusion of the corresponding retroperitoneal lymph nodes (Figure 2C).

The patient underwent robot-assisted left partial nephrectomy (RAPN) with concurrent laparoscopic para-aortic lymph node dissection (console time, 4 h 58 min; estimated blood loss, 50 mL). Histopathological examination confirmed clear cell RCC (World Health Organization/International Society of Urological Pathology grade 1>2, pT1a, 22 mm, ly0, v1), establishing a final diagnosis of RCC with testicular metastasis (pT1aN1M1, International Metastatic Renal Cell Carcinoma Database Consortium intermediate risk).

**Figure 3:**
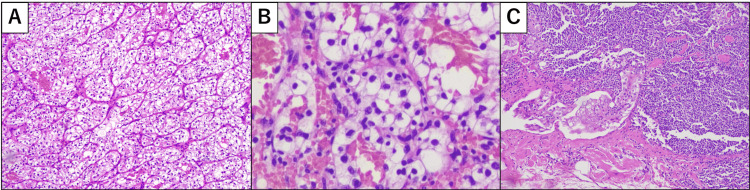
Histopathological findings of primary renal cell carcinoma and the para-aortic lymph nodes. A: Hematoxylin and eosin (H&E) staining following robot-assisted left partial nephrectomy and laparoscopic para-aortic lymph node dissection shows a clear cell RCC (×100). B: Higher magnification shows tumor cells with clear cytoplasm, a low nuclear-to-cytoplasm ratio, and mild nuclear irregularity (H&E, ×400). C: Metastatic involvement of the para-aortic lymph nodes with a characteristic small nest is shown (H&E, ×100).

Systemic therapy with pembrolizumab (480 mg every six weeks) was initiated two months after RAPN. However, the treatment was discontinued after two cycles owing to grade 2 glucose intolerance and hypothyroidism secondary to destructive thyroiditis (Common Terminology Criteria for Adverse Events, version 5) (Figure 3). Follow-up CT seven months after RAPN revealed disease progression with new mediastinal and internal thoracic lymphadenopathies in addition to the previously detected retroperitoneal lymphadenopathies. Second-line therapy with cabozantinib was initiated nine months post-RAPN but was discontinued after one month owing to elevated KL-6 levels and worsening hypothyroidism. The patient ultimately died of progressive metastatic disease 21 months after RAPN. 

## Discussion

The relationship between RCC size and metastatic potential is clinically intriguing. While small renal masses (≤4 cm) have an excellent prognosis, 1.1-6.2% of RCCs <3.0 cm in diameter present with distant metastases at the initial diagnosis, which significantly worsens survival outcomes [4-7]. In a multi-institutional study, synchronous metastases were observed in 4.2% of 2,197 small renal masses including T1a tumors [8]. In a large cohort study of 2,651 T1a RCCs, the 5-year cancer-specific survival rate was significantly lower in the presence vs. absence of synchronous metastases (42% vs. 96%, p <0.001) [9]. Thus, despite their rarity, metastatic small renal masses significantly impact patient outcomes.

The biological mechanisms underlying the aggressive behavior of small RCCs remain poorly understood. However, several studies suggest that molecular markers such as AMP-activated kinase (AMPK) and fatty acid production may better predict metastatic potential rather than tumor size alone [10].

The estimated incidence rate of malignant metastasis to the testis is 0.3%-3.6% [11](PMID: 28559825). And it has a distinct distribution pattern, with the primary tumor originating from the prostate (35%), lungs (19%), colon (9%) or kidney (7%) or a melanoma (9%) [12]. A comprehensive literature review uncovered 31 documented cases [13]. 

Two principal protective mechanisms likely account for the inherent resistance of the testicular microenvironment to metastatic seeding: the physiologically low scrotal temperature and the immunologically privileged environment maintained by the blood-testis barrier, which is established by specialized Sertoli cells [14]. The pathophysiological mechanisms underlying the testicular metastasis of an RCC appear to be site-dependent. Ipsilateral testicular involvement typically results from retrograde venous dissemination via the testicular vein, whereas contralateral metastasis, as demonstrated in our case, utilizes alternative metastatic pathways [3]. Potential routes of contralateral spread include the Batson venous plexus, a unique valveless network of paravertebral veins connecting pelvic organs, thoracic vessels, and intraspinal structures. Owing to its distinctive anatomy, this system lacks conventional valvular mechanisms and facilitates bidirectional flow and non-lymphatic metastatic dissemination [15].

Among 31 cases of renal cell carcinoma with testicular metastasis reported in a comprehensive literature review, only three were classified as T1a, and two of the three cases exhibited contralateral testicular involvement (Table 1) [13]. Our case is particularly significant because it is the first report (to the best of our knowledge) of testicular metastasis as the initial clinical manifestation, leading to the subsequent identification of a contralateral T1a RCC [14,16,17].

**Table 1:** Clinical characteristics of pT1a renal cell carcinomas that metastasized to the testis. RCC: Renal cell carcinoma, mRCC: metastatic renal cell carcinoma, LVI: lymphovascular invasion, N/A: not applicable.

Case No.	References	Age of testicular mRCC diagnosis (Years)	Histopathological type	Grading in the Furman scale	Testicular mRCC side in relation to kidney primary RCC side	LVI in kidney tumor	Other sites of metastases at the time of testicular mRCC diagnosis	Testicular metastasis as the first clinical manifestation of RCC	Follow-up after orchiectomy (month)	Outcome
1	Wang et al. 2020 [16]	76	Clear cell	1	Contralateral	No	No	No	93	Alive with disease
2	Turco et al. 2021 [17]	73	Clear cell	1	Ipsilateral	N/A	Lung, liver, pancreas	No	26	Dead of disease
3	Thomson et al. 2023 [14]	80	Clear cell	2	Contralateral	N/A	No	No	N/A	
4	This case 2025	64	Clear cell	1	Contralateral	Yes	Retroperitoneal lymph node	Yes	24	Dead of disease

The distinctive features of our case necessitated a careful diagnostic approach. The exceptional rarity of contralateral testicular metastasis from a small renal tumor, combined with non-characteristic histopathological findings, prompted us to perform concurrent para-aortic lymph node dissection and RAPN. Despite only modest lymphadenopathy, this decision was crucial for a definitive diagnosis and appropriate staging.

This case contributes several valuable insights to the existing literature. First, it challenges the presumed association between tumor RCC size and metastatic potential. Second, it shows that unusual metastatic patterns may precede the diagnosis of a primary malignancy. Third, it emphasizes the importance of considering metastatic disease even in cases of small renal masses.

From a clinical perspective, this case highlights a critical diagnostic principle: although testicular masses in elderly patients typically suggest primary testicular neoplasms, differential diagnoses, including metastatic disease from unexpected primary sites, should be comprehensively considered. A systematic diagnostic approach incorporating advanced imaging modalities and thorough histopathological analysis is essential for accurate diagnosis and optimal therapeutic planning.

## Conclusions

We reported the first documented case of a pT1a RCC initially presenting as a contralateral testicular metastasis. Testicular masses are usually treated with orchiectomy. However, pathologic results of testicular tumors alone may not lead to a definitive diagnosis as in this case. Physicians should consider the possibility of testicular metastasis from other primary cancer sites when diagnosing a testicular mass.
